# Isospectrals of non-uniform Rayleigh beams with respect to their uniform counterparts

**DOI:** 10.1098/rsos.171717

**Published:** 2018-02-14

**Authors:** Srivatsa Bhat K, Ranjan Ganguli

**Affiliations:** Department of Aerospace Engineering, Indian Institute of Science, Bangalore 560012, India

**Keywords:** Rayleigh beam, isospectral, non-uniform beam, finite-element method

## Abstract

In this paper, we look for non-uniform Rayleigh beams isospectral to a given uniform Rayleigh beam. Isospectral systems are those that have the same spectral properties, i.e. the same free vibration natural frequencies for a given boundary condition. A transformation is proposed that converts the fourth-order governing differential equation of non-uniform Rayleigh beam into a uniform Rayleigh beam. If the coefficients of the transformed equation match with those of the uniform beam equation, then the non-uniform beam is isospectral to the given uniform beam. The boundary-condition configuration should be preserved under this transformation. We present the constraints under which the boundary configurations will remain unchanged. Frequency equivalence of the non-uniform beams and the uniform beam is confirmed by the finite-element method. For the considered cases, examples of beams having a rectangular cross section are presented to show the application of our analysis.

## 1. Introduction

Non-uniform beams are used to mathematically model many important mechanical, aerospace and naval engineering structures. To accurately model long and slender beams, the Euler–Bernoulli beam theory is sufficient; whereas, for short and thick beams, and for accurate frequency prediction of the higher modes of vibration, the Bresse–Timoshenko beam theory is more widely used. A relatively ‘simpler theory’ was developed by Lord Rayleigh [1] before the Timoshenko beam theory came into existence which includes the rotary inertia effect but does not take into account the shear deformation [2–5]. The governing equation of Rayleigh beams is a single fourth-order differential equation in a single variable as opposed to the coupled differential equations in two variables (harmonic vibrations) for the Timoshenko beams. The Rayleigh beam theory also predicts the natural frequencies and mode shapes more accurately than the Euler–Bernoulli beam theory, without going into the mathematical complexities of the Timoshenko beam theory.

Inverse problems are an important class of problems in vibrating systems which involve finding material and geometric properties from known modal parameters and reconstruction of a beam from its spectral data [6]. Multiple beams can have same spectra for a given boundary condition. The existence of systems that have the same frequencies for a given boundary condition but have different material and geometric properties is of great interest in mechanics.

Finding isospectral systems is an important subclass of inverse problems. This involves finding beams which have same spectra as that of a known beam. Isospectral Euler–Bernoulli beams with continuous density and rigidity functions were analysed by Gottlieb [7]. Seven different classes were found to be analytically solvable and isospectral to a homogeneous beam, and corrections to the transformation used by Barcilon [8] were made. Subramanian & Raman [9] generalized the transformation to obtain isospectral systems for all taper powers. Ghanbari [10] found 12 classes of isospectral beams by factoring the fourth-order beam operator into two second-order differential operators for four different boundary conditions. Gladwell & Morassi [11] considered a specific class of beams where the product of stiffness and mass per unit length is constant. Boundary conditions being any combination of pinned and sliding, they obtained a closed form expression for beams isospectral to a given beam. The special class of beams was equivalent to a string and Darboux lemma was used to reduce the string equation to Sturm–Liouville canonical form. In [12,13], a procedure for obtaining real densities of circular membranes that are isospectral to a given uniform circular membrane under fixed and free boundary conditions is introduced by Gottlieb. In [12], it is shown that membranes isospectral to radial density membranes no longer possess radial symmetry. Kambampati & Ganguli [14] extended the analysis to find non-uniform rotating beams isospectral to a given uniform rotating beam. Kambampati *et al.* [15,16] found non-rotating beams isospectral to rotating uniform beams and rotating beams isospectral to axially loaded non-rotating uniform beams. Kambampati & Ganguli [17,18] found non-uniform beams and stiff springs isospectral to axially loaded uniform beams and piano strings, and non-rotating beams isospectral to tapered rotating beams. In their study, they used Barcilon–Gottlieb transformation to convert the fourth-order governing equation of one kind to the required one. Then they validated the results using the finite-element method (FEM) and provided examples of isospectral rectangular cross-section beams as the application of their analysis.

In this paper, we extend the analysis to Rayleigh beams. We use a transformation to convert the non-dimensional non-uniform Rayleigh beam to a uniform Rayleigh beam, from the (*x*,*W*) frame of reference to a hypothetical (*z*,*U*) frame of reference. If the material and geometric properties of the beam are specific chosen functions of the two introduced auxiliary variables, then the transformation will be achieved and, if the coefficients of the transformed equation match with the uniform one, then the equivalence is established. Four specific cases are considered for solving a pair of coupled ODEs and we arrive at the closed form solutions for the mass per unit length, mass moment of inertia and bending stiffness variations of the non-uniform beam which is isospectral to the given uniform beam. Also, we present the constraints under which the boundary conditions can remain unchanged. Examples of beams having a rectangular cross section are presented to show the application of our analysis.

## 2. Mathematical formulation

The equation governing the free vibrations of a non-uniform, inhomogeneous Rayleigh beam of length *L* with angular frequency *ω* and transverse displacement *W*(*x*) is

2.1 $$\frac{d^{2}}{dX^{2}}\left(EI\frac{d^{2}W}{dX^{2}}\right)+\frac{d}{dX}\left(\rho I\omega^{2}\frac{dW}{dX}\right)-\rho A\omega^{2}W=0,\;0\leq X\leq L,$$

where *ρA* is the mass per unit length, *EI* is the bending rigidity and *ρI* is the mass moment of inertia per unit length. Here, *E*, *G* and *ρ* denote Young’s modulus, the shear modulus and the mass density, respectively; *A* and *I* denote the area and the area moment of inertia of the cross section, respectively. For a given uniform beam, flexural stiffness *EI*(*X*)=*E*_0_*I*_0_, mass per unit length *ρA*(*X*)=*ρ*_0_*A*_0_ and mass moment of inertia per unit length *ρI*(*X*)=*ρ*_0_*I*_0_. We introduce non-dimensional variables *f*, *g*, *m* and *x* as

$$f(x)=\frac{EI(x)}{E_{0}I_{0}},\;g(x)=\frac{\rho I(x)}{\rho_{0}I_{0}},\;m(x)=\frac{\rho A(x)}{\rho_{0}A_{0}}\;\mathrm{and}\;x=\frac{X}{L}.$$

Then, equation (2.1) can be written in non-dimensional form as

2.2 $$\frac{d^{2}}{dx^{2}}\left(f(x)\frac{d^{2}W}{dx^{2}}\right)+\eta^{2}{\left(\frac{r_{0}}{L}\right)}^{2}\frac{d}{dx}\left(g(x)\frac{dW}{dx}\right)-m(x)\eta^{2}W=0,\;0\leq x\leq 1,$$

where *η* (non-dimensional natural frequency) and *r*_0_ are given by

$$r_{0}=\sqrt{\frac{I_{0}}{A_{0}}}\;\mathrm{and}\;\eta^{2}=\omega^{2}\left(\frac{\rho_{0}A_{0}L^{4}}{E_{0}I_{0}}\right).$$

For reference, a uniform Rayleigh beam will be defined with displacement function *V* (*z*) that satisfies the equation

2.3 $$\left(\frac{d^{4}V}{dz^{4}}\right)+\eta^{2}{\left(\frac{r_{0}}{L}\right)}^{2}\;\frac{d^{2}V}{dz^{2}}-\eta^{2}V=0,\;0\leq z\leq 1.$$

The second term on the left-hand side of the above equation is present in the Rayleigh formulation and will not appear in the Euler–Bernoulli formulation [14]. The non-dimensional governing equation (i.e. equation (2.2)), which is in the (*x*,*W*) frame, can be transformed into the (*z*,*U*) frame, using the following transformations [7]:

2.4 W(x(z))=q(z)U(z)

and

2.5 $$x=\int_{0}^{z}\frac{1}{p(z)}\;dz,$$

where *p* and *q* are the auxiliary variables involved in the transformation.

Equation (2.5) implies that

2.6 $$\begin{array}{rl} z & =\int_{0}^{x}p(z(x))\;dx\Rightarrow \frac{d}{dx}=p(z)\frac{d}{dz}\Rightarrow \frac{1}{p(z)}=\frac{dx}{dz}\\ \;\text{and}\;x & =0\Leftrightarrow z=0\;\text{and}\;x=1\Leftrightarrow z=z_{0} \end{array}\}$$

where *z*_0_ is defined such that the above relation holds.

Here *f*, *g* and *m* are functions of *p* and *q* and are chosen in such a way as to transform equation (2.2) to the desired equation (equation (2.8)), and are given by

2.7 $$f=\frac{1}{p^{3}q^{2}},\;g=\frac{1}{pq^{2}}\;\mathrm{and}\;m=\frac{p({\left(r_{0}/L\right)}^{2}qq^{\prime \prime }-2(r_{0}/L){}^{2}q^{\prime 2}+q^{2})}{q^{4}}.$$

Substituting equation (2.7) into equation (2.2) and applying the transformation, we have

2.8 $$\begin{array}{rl} & \frac{p}{q}\frac{d^{4}U}{dz^{4}}+\frac{1}{pq^{3}}\frac{d^{2}U}{dz^{2}}\left(2pq\left(2p\frac{d^{2}q}{dz^{2}}-\frac{dp}{dz}\frac{dq}{dz}\right)+q^{2}\left(p\frac{d^{2}p}{dz^{2}}-2{\left(\frac{dp}{dz}\right)}^{2}\right)-6p^{2}{\left(\frac{dq}{dz}\right)}^{2}\right)\\ & \;+\frac{1}{p^{2}q^{4}}\frac{dU}{dz}(2p^{2}q\frac{dq}{dz}\left(\frac{dp}{dz}\frac{dq}{dz}-8p\frac{d^{2}q}{dz^{2}}\right)+2pq^{2}\left(-p\frac{dp}{dz}\frac{d^{2}q}{dz^{2}}+p\left(2p\frac{d^{3}q}{dz^{3}}-\frac{d^{2}p}{dz^{2}}\frac{dq}{dz}\right)+{\left(\frac{dp}{dz}\right)}^{2}\frac{dq}{dz}\right)\\ & \;+q^{3}\left(p^{2}\frac{d^{3}p}{dz^{3}}-5p\frac{d^{2}p}{dz^{2}}\frac{dp}{dz}+4{\left(\frac{dp}{dz}\right)}^{3}\right)+12p^{3}{\left(\frac{dq}{dz}\right)}^{3})+\frac{1}{p^{2}q^{4}}U(6p^{2}{\left(\frac{dq}{dz}\right)}^{2}\left(p\frac{d^{2}q}{dz^{2}}+\frac{dp}{dz}\frac{dq}{dz}\right)\\ & \;-2pq\left(2p\frac{dp}{dz}\frac{d^{2}q}{dz^{2}}\frac{dq}{dz}+p\left(2p\frac{d^{3}q}{dz^{3}}\frac{dq}{dz}+2\frac{d^{2}p}{dz^{2}}{\left(\frac{dq}{dz}\right)}^{2}+p{\left(\frac{d^{2}q}{dz^{2}}\right)}^{2}\right)-3{\left(\frac{dp}{dz}\right)}^{2}{\left(\frac{dq}{dz}\right)}^{2}\right)\\ & \;+q^{2}\left(-2p{\left(\frac{dp}{dz}\right)}^{2}\frac{d^{2}q}{dz^{2}}-5p\frac{d^{2}p}{dz^{2}}\frac{dp}{dz}\frac{dq}{dz}+p^{2}\left(p\frac{d^{4}q}{dz^{4}}+\frac{d^{3}p}{dz^{3}}\frac{dq}{dz}+\frac{d^{2}p}{dz^{2}}\frac{d^{2}q}{dz^{2}}\right)+4{\left(\frac{dp}{dz}\right)}^{3}\frac{dq}{dz})\right)\\ & \;+\eta^{2}\left(\frac{d^{2}U}{dz^{2}}{\left(\frac{r_{0}}{L}\right)}^{2}\frac{p}{q}-\frac{p}{q}U\right)=0. \end{array}$$

If *A* and *B* are given by

2.9 $$A=-\frac{2(dp/dz)(dq/dz)}{pq}+\frac{d^{2}p/dz^{2}}{p}-\frac{2(dp/dz{)}^{2}}{p^{2}}+\frac{4(d^{2}q/dz^{2})}{q}-\frac{6(dq/dz{)}^{2}}{q^{2}}$$

and

2.10 $$\begin{array}{rl} B & =\frac{4p^{\prime 3}q^{\prime }}{p^{3}q}+\frac{6p^{\prime 2}q^{\prime 2}}{p^{2}q^{2}}+\frac{6p^{\prime }q^{\prime 3}}{pq^{3}}-\frac{5p^{\prime }q^{\prime }p^{\prime \prime }}{p^{2}q}-\frac{4q^{\prime 2}p^{\prime \prime }}{pq^{2}}-\frac{2q^{\prime \prime }}{q^{2}}-\frac{2p^{\prime 2}q^{\prime \prime }}{p^{2}q}\\ & \;-\frac{4p^{\prime }q^{\prime }q^{\prime \prime }}{pq^{2}}+\frac{6q^{\prime 2}q^{\prime \prime }}{q^{3}}+\frac{p^{\prime \prime }q^{\prime \prime }}{pq}+\frac{q^{\prime }p^{\prime \prime \prime }}{pq}-\frac{4q^{\prime }q^{\prime \prime \prime }}{q^{2}}+\frac{q^{⁗}}{q}, \end{array}$$

then equation (2.8) can be rewritten as

2.11 $$\left(\frac{d^{4}U}{dz^{4}}\right)+\frac{d}{dz}\left(A\frac{dU}{dz}\right)+BU+\eta^{2}{\left(\frac{r_{0}}{L}\right)}^{2}\frac{d^{2}U}{dz^{2}}-\eta^{2}U=0,\;0\leq z\leq z_{0}.$$

If *A*,*B*=0, then equation (2.3) is identical to equation (2.11), thus transforming the non-uniform equation to a uniform one.

Thus, the non-uniform beams are isospectral to a given uniform beam if

2.12 A=0andB=0.

These equations are coupled fourth-order ODEs and are difficult to solve. However, for four special cases: (i) *q*=*q*_0_, a constant, (ii) *p*(d*q*/d*z*)=*k*, a constant, (iii) *pq*^2^=*k*, a constant, and (iv) *p*=*p*_0_, a constant, we show that the coupled ODEs can be solved analytically.

### 2.1. Case 1: *q*=*c*_1_, a constant

When *q*=*c*_1_, *B*=0 is automatically satisfied. Setting *A*=0 yields

2.13 $$p\frac{d^{2}p}{dz^{2}}-2{\left(\frac{dp}{dz}\right)}^{2}=0.$$

Solving for *p*, we have

2.14 $$p(z)=\frac{1}{\beta +\alpha z},$$

where *α* and *β* are arbitrary constants in the general solution.

Substituting equation (2.14) into equation (2.5) and then solving for *z*, we have

2.15 $$z=\left\{\frac{\sqrt{\beta^{2}+2\alpha x}-\beta }{\alpha },-\frac{\beta +\sqrt{\beta^{2}+2\alpha x}}{\alpha }\right\}.$$

For positive *α* and *β*, we require *z* to be positive, thus

2.16 $$z=\frac{\sqrt{\beta^{2}+2\alpha x}-\beta }{\alpha }.$$

Substituting *p*(*z*), *q*(*z*) and equation (2.16) into equation (2.7), we have

2.17 $$\begin{array}{rl} \left\{f(x),g(x),m(x)\right\} & =\{\frac{{\left(\beta +(1/2)\left(\sqrt{\beta^{2}+4\alpha x}-\beta \right)\right)}^{3}}{c_{1}^{2}},\frac{\beta +(1/2)\left(\sqrt{\beta^{2}+4\alpha x}-\beta \right)}{c_{1}^{2}},\\ & \;\frac{1}{c_{1}^{2}\left(\beta +(1/2)\left(\sqrt{\beta^{2}+4\alpha x}-\beta \right)\right)}\}. \end{array}$$

**Figure 1.**
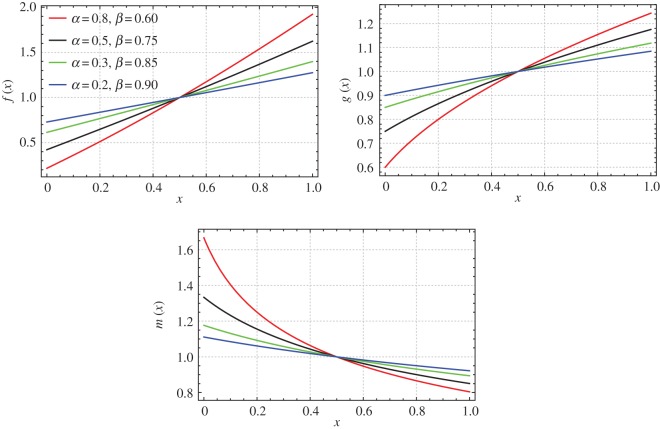
Mass, bending stiffness and mass moment of inertia functions of a non-uniform beam isospectral to a uniform beam (Case 1: *β*={0.60,0.75,0.85,0.90}, *c*_1_=1 and *r*_0_/*L*=0.09).

If *z*_0_=1⇔*x*_0_=1, then *α* and *β* are related by the following expression: *α*=2(1−*β*). For *β*={0.6,0.75,0.85,0.9}, *c*_1_=1 and *r*_0_/*L*=0.09 we plot the mass, stiffness and mass moment of inertia functions of the non-uniform beams which are isospectral to a uniform beam (figure 1).

We apply our analysis to beams having a rectangular cross section. The non-dimensional breadth (*b*) and height (*h*) profiles of the cross sections are related to the mass and mass moment of inertia of the beams by the following relation:

2.18 $$\begin{array}{rl} m(x) & =b(x)h(x);\;g(x)=b(x)h{\left(x\right)}^{3} \end{array}$$

2.19 $$\begin{array}{r} \Rightarrow \;b(x)=\sqrt{\frac{m{\left(x\right)}^{3}}{g(x)}};\;h(x)=\sqrt{\frac{g(x)}{m(x)}}. \end{array}$$

**Figure 2.**
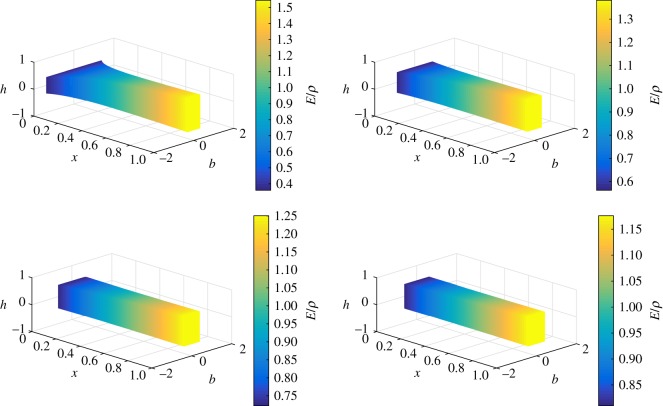
Breadth, height and ratio of modulus and density distributions of non-uniform beams isospectral to a uniform beam (Case 1: *β*={0.60,0.75,0.85,0.90}, *c*_1_=1 and *r*_0_/*L*=0.09).

Also, *f*(*x*)/*g*(*x*) corresponds to the ratio *E*/*ρ*. Therefore, using the *m*(*x*), *g*(*x*) and *f*(*x*) functions, we can derive the *b*, *h* and *E*/*ρ* profiles of the rectangular beams. These *b*, *h* and *E*/*ρ* profiles of the non-uniform beams, which are isospectral to a uniform beam, are plotted in figure 2. These results show that the height, breadth and the ratio of modulus and density vary along the *x*-axis in such a way as to force the natural frequencies to remain identical to that of the uniform beam.

### 2.2. Case 2: *pq*_*z*_=*k*, a constant

If *p*(d*q*/d*z*)=*k*, then *B*=0 is automatically satisfied (eqn (2.22a) in [7]). Setting A = 0 yields

2.20 $$\frac{6q^{\prime \prime }(z)}{q(z)}-\frac{6q^{\prime }{\left(z\right)}^{2}}{q{\left(z\right)}^{2}}-\frac{q^{\left(3\right)}(z)}{q^{\prime }(z)}=0.$$

Solving for *q*(*z*), then obtaining *p*(*z*) from the constraint, we have

2.21 $$\begin{array}{rl} q(z) & =\frac{1}{\alpha +\beta z+\gamma z^{2}}\\ \;\text{and}\;p(z) & =-\frac{k{\left(\alpha +\gamma z^{2}+\beta z\right)}^{2}}{\beta +2\gamma z}, \end{array}\}$$

where *α*, *β* and *γ* are arbitrary constants in the general solution.

Substituting *p*(*z*) from equation (2.21) into equation (2.5), we have

2.22 $$x=\frac{1}{k(\alpha +z(\beta +\gamma z))}-\frac{1}{\alpha k}$$

and

2.23 $$\begin{array}{rl} z & =\{\frac{-\beta -\sqrt{{\left(\beta +\alpha \beta kx\right)}^{2}-4\alpha^{2}kx(\gamma +\alpha \gamma kx)}+\alpha \beta (-k)x}{2(\gamma +\alpha \gamma kx)},\\ & \;\frac{-\beta +\sqrt{{\left(\beta +\alpha \beta kx\right)}^{2}-4\alpha^{2}kx(\gamma +\alpha \gamma kx)}+\alpha \beta (-k)x}{2(\gamma +\alpha \gamma kx)}\}. \end{array}$$

Substituting *p*(*z*), *q*(*z*) and second expression of equation (2.23) into equation (2.7) with *k*=−1, we have

2.24 $$\begin{array}{rl} f(x) & =\frac{(1-\alpha x)(-(\alpha x-1)(4\alpha^{2}\gamma x+\beta^{2}(1-\alpha x)){)}^{3/2}}{\alpha^{4}},\\ g(x) & =\frac{\sqrt{-(\alpha x-1)(4\alpha^{2}\gamma x+\beta^{2}(1-\alpha x))}}{1-\alpha x}\\ \;\text{and}\;m(x) & =-\frac{\alpha^{3}(\alpha +2\gamma r_{0}^{2}(\alpha x-1))}{{\left(\alpha x-1\right)}^{3}\sqrt{-(\alpha x-1)(4\alpha^{2}\gamma x+\beta^{2}(1-\alpha x))}}. \end{array}\}$$

**Figure 3.**
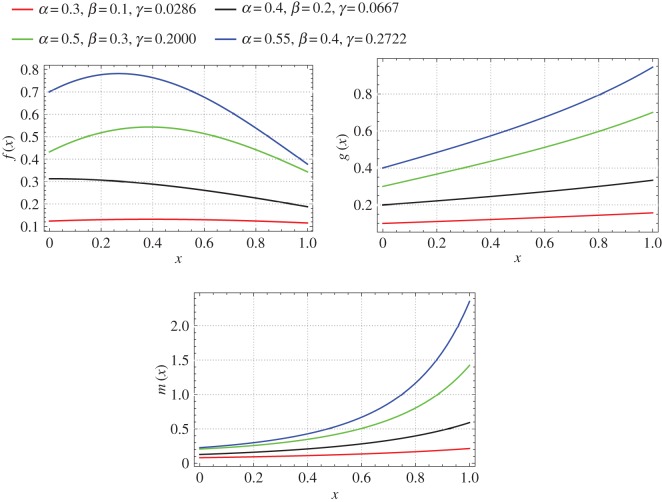
Mass, bending stiffness and mass moment of inertia functions of a non-uniform beam isospectral to a uniform beam (Case 2: *α*={0.3,0.4,0.5,0.55}, *β*={0.1,0.2,0.3,0.4}, *k*=−1, and *r*_0_/*L*=0.09).

If *z*_0_=1⇔*x*_0_=1, then *α*, *β* and *γ* are related by the expression: *γ*=(−*β*+*α*^2^(−*k*)−*αβk*)/(*αk*+1). For *α*={0.3,0.4,0.5,0.55}, *β*={0.1,0.2,0.3,0.4}, *k*=−1 and *r*_0_/*L*=0.09 we plot the mass, stiffness and mass moment of inertia functions of the non-uniform beams which are isospectral to a uniform beam as shown in figure 3.

**Figure 4.**
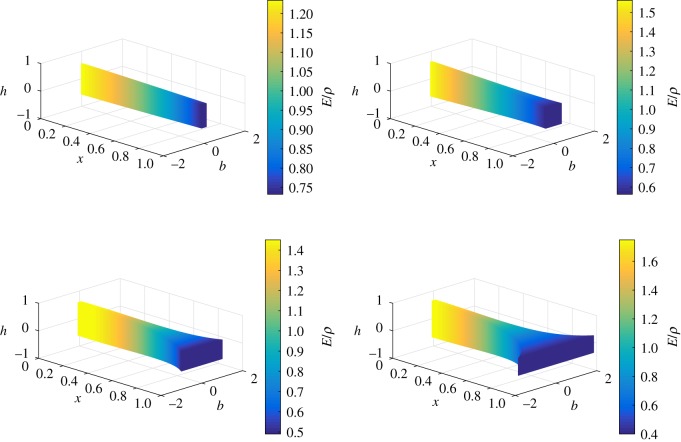
Breadth, height and ratio of modulus and density distributions of non-uniform beams isospectral to a uniform beam (Case 2: *α*={0.3,0.4,0.5,0.55}, *β*={0.1,0.2,0.3,0.4}, *k*=−1, and *r*_0_/*L*=0.09).

Applying our analysis to beams having a rectangular cross section, the *b*, *h* and *E*/*ρ* profiles of the non-uniform beams which are isospectral to the given uniform beam are shown in figure 4.

### 2.3. Case 3: *pq*^2^=*c*, a constant

If *pq*^2^=*c*, then substituting *p* into *A*, and setting it to zero and simplifying, we get *p*(d*q*/d*z*)=const., which automatically satisfies *B*=0. From the above two conditions, we have

2.25 $$\frac{dq}{dz}\frac{1}{q^{2}}=k.$$

Solving for *q*(*z*) and then obtaining *p*(*z*) from the constraint, we have

2.26 $$\begin{array}{rl} q(z) & =\frac{1}{-\alpha -kz}\\ \;\text{and}\;p(z) & =c{\left(-\alpha -kz\right)}^{2}. \end{array}\}$$

Substituting *p*(*z*) from equation (2.26) into equation (2.5) and then solving for *z*, we arrive at the expression

2.27 $$z=\frac{\alpha^{2}cx}{\alpha cx+1}.$$

Substituting *p*(*z*), *q*(*z*) and equation (2.27) into equation (2.7) with *k*=−1, we have

2.28 $$\{f(x),g(x),m(x)\}=\left\{\frac{{\left(\alpha cx+1\right)}^{4}}{\alpha^{4}c^{3}},\frac{1}{c},\frac{\alpha^{4}c}{{\left(\alpha cx+1\right)}^{4}}\right\}.$$

**Figure 5.**
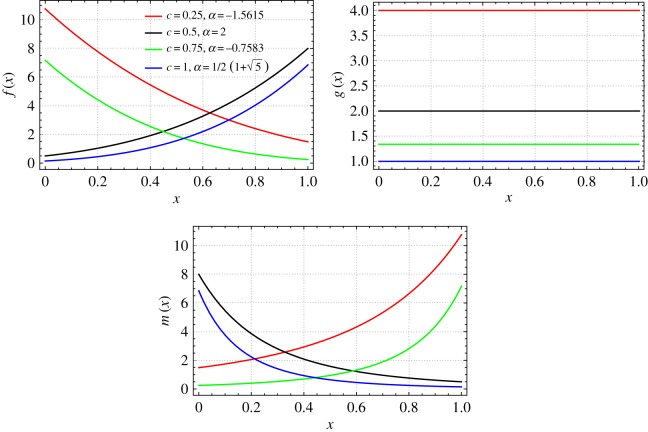
Mass, bending stiffness and mass moment of inertia functions of a non-uniform beam isospectral to a uniform beam (Case 3: *c*={0.25,0.5,0.75,1}, $\alpha =\{-1.56155,2,-0.758306,\frac{1}{2}\left(\sqrt{5}+1\right)\}$, *k*=−1 and *r*_0_/*L*=0.09).

**Figure 6.**
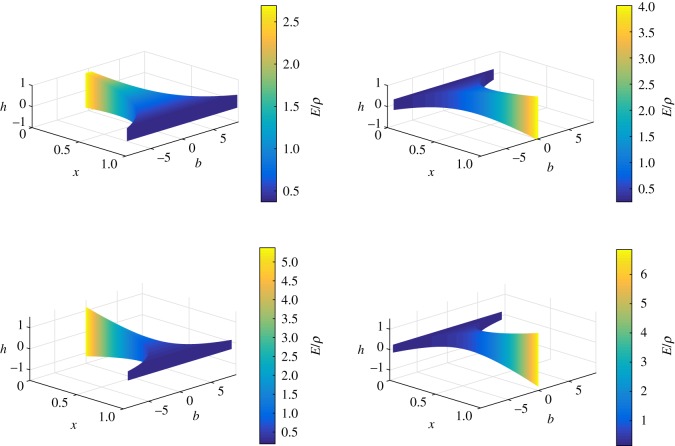
Breadth, height and ratio of modulus and density distributions of non-uniform beams isospectral to a uniform beam (Case 3: *c*={0.25,0.5,0.75,1}, $\alpha =\left\{-1.56155,2,-0.758306,\frac{1}{2}\left(\sqrt{5}+1\right)\right\}$, *k*=−1 and *r*_0_/*L*=0.09).

If *z*_0_=1⇔*x*_0_=1, then *α* and *c* are related by the expression: $\alpha =\{-(\sqrt{c}\sqrt{c+4}-c)/2c,(c+\sqrt{c}\sqrt{c+4})/2c\}$. If *c*={0.25,0.5,0.75,1} and if *α* is obtained alternatively from its expressions, then the values of *α* obtained are as follows: $\alpha =\left\{-1.56155,2,-0.758306,\frac{1}{2}\left(\sqrt{5}+1\right)\right\}$. For *k*=−1, *r*_0_/*L*=0.09, we plot the mass, stiffness and mass moment of inertia functions of the non-uniform beams which are isospectral to a uniform beam as shown in figure 5. The *b*, *h* and *E*/*ρ* profiles of the non-uniform beams which are isospectral to the given uniform beam are shown in figure 6.

### 2.4. Case 4: *p*=*k*, a constant

If *p*=*k*, then *z*=*kx* and, if *A*=0, then

2.29 $$\frac{4(d^{2}q/dz^{2})}{q}-\frac{6{\left(dq/dz\right)}^{2}}{{\left(q\right)}^{2}}=0.$$

Solving for *q*(*z*), we have

2.30 $$q(z)=\frac{1}{{\left(\beta +\alpha z\right)}^{2}}.$$

Substituting *p* and *q*(*z*) into equation (2.7), we obtain

2.31 $$\{\;f(x),g(x),m(x)\}=\left\{\frac{{\left(\beta +\alpha kx\right)}^{4}}{k^{3}},\frac{{\left(\beta +\alpha kx\right)}^{4}}{k},k{\left(\beta +\alpha kx\right)}^{8}\;\left(\frac{1}{{\left(\beta +\alpha kx\right)}^{4}}-\frac{2\alpha^{2}r_{0}^{2}}{{\left(\beta +\alpha kx\right)}^{6}}\right)\right\}.$$

**Figure 7.**
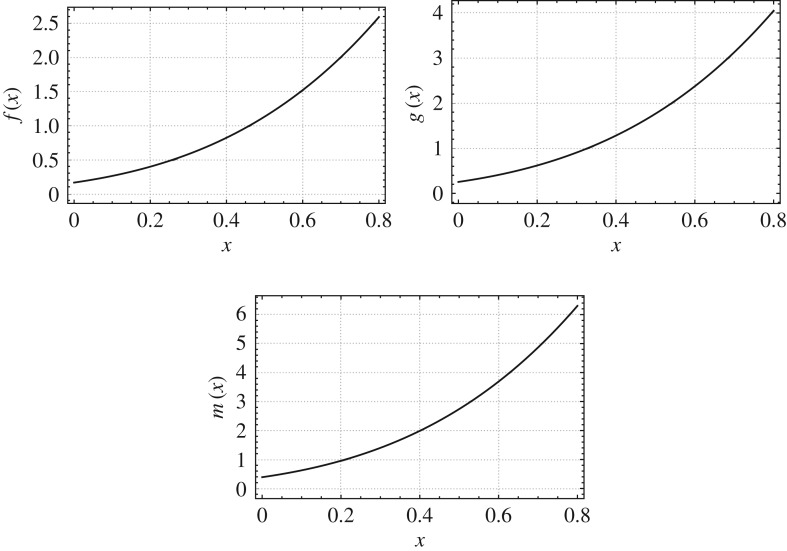
Mass, bending stiffness and mass moment of inertia functions of a non-uniform beam isospectral to a uniform beam (Case 4: *k*={0.75},*r*_0_/*L*=0.09,*α*= 0.75 and *β*=0.75).

**Figure 8.**
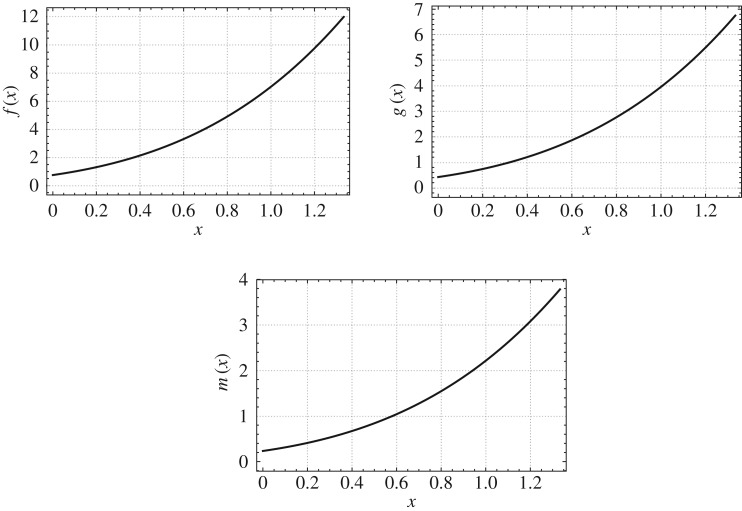
Mass, bending stiffness and mass moment of inertia functions of a non-uniform beam isospectral to a uniform beam (Case 4: *k*={1.25},*r*_0_/*L*=0.09,*α*= 0.75 and *β*=0.75).

**Figure 9.**
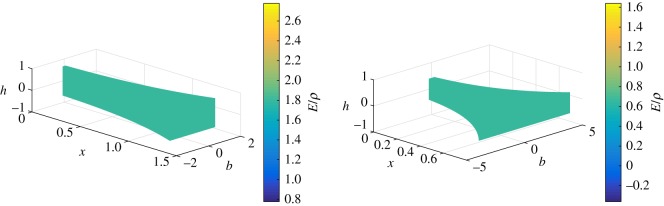
Breadth, height and ratio of modulus and density distributions of non-uniform beams isospectral to a uniform beam (Case 4: *k*={1.25,0.75},*r*_0_/*L*= 0.09,*α*=0.75 and *β*=0.75).

For *k*={0.75,1.25},*r*_0_/*L*=0.09,*α*=0.75 and *β*=0.75, we plot the mass, stiffness and mass moment of inertia functions of the non-uniform beams which are isospectral to the uniform beam as shown in figures 7 and 8. The *b*, *h* and *E*/*ρ* profiles of the non-uniform beams which are isospectral to the given uniform beam are shown in figure 9.

## 3. Boundary conditions

While applying the transformation to the differential equation, the boundary conditions also get transformed. The clamped boundary condition remains invariant, but for certain specific cases, other boundary conditions can also remain invariant. We present various conditions under which the boundary conditions remain invariant (table 1). The above transformation transforms the derivatives *W*_*x*_,*W*_*xx*_,*W*_*xxx*_ in terms of *U*_*z*_,*U*_*zz*_,*U*_*zzz*_ as follows:

3.1 $$\begin{array}{r} W=qU, \end{array}$$

3.2 $$\begin{array}{r} \frac{dW}{dx}=p\left(q\frac{dU}{dz}+U\frac{dq}{dz}\right), \end{array}$$

3.3 $$\begin{array}{r} \frac{d^{2}W}{dx^{2}}={\left(p\right)}^{2}q\frac{d^{2}U}{dz^{2}}+p\frac{dU}{dz}\left(2p\frac{dq}{dz}+q\frac{dp}{dz}\right)+pU\left(p\frac{d^{2}q}{dz^{2}}+\frac{dp}{dz}\frac{dq}{dz}\right) \end{array}$$

3.4 $$\begin{array}{rl} \;\text{and}\;\frac{d^{3}W}{dx^{3}} & ={\left(p\right)}^{3}q\frac{d^{3}U}{dz^{3}}+3{\left(p\right)}^{2}\frac{d^{2}U}{dz^{2}}\left(p\frac{dq}{dz}+q\frac{dp}{dz}\right)+p\frac{dU}{dz}(3p\left(p\frac{d^{2}q}{dz^{2}}+2\frac{dp}{dz}\frac{dq}{dz}\right)\\ & \;+q\left(p\frac{d^{2}p}{dz^{2}}+{\left(\frac{dp}{dz}\right)}^{2}\right))+pU\left(3p\frac{dp}{dz}\frac{d^{2}q}{dz^{2}}+p\left(p\frac{d^{3}q}{dz^{3}}+\frac{d^{2}p}{dz^{2}}\frac{dq}{dz}\right)+{\left(\frac{dp}{dz}\right)}^{2}\frac{dq}{dz}\right). \end{array}$$

**Table 1.** A summary of the boundary conditions, and the cases under which they are preserved.

boundary condition		condition on the auxiliary variables
clamped	*W*=0=*W*_*x*_	Cases (i)–(iv)
pinned	*W*=0=*W*_*xx*_	Case (iii)
free	$W_{xx}=0=\frac{d}{dx}\left(f(x)\frac{d^{2}W}{dx^{2}}\right)+\eta^{2}{\left(\frac{r_{0}}{L}\right)}^{2}g(x)\frac{dW}{dx}$	*p* and *q*=*const*.
antiresonant(i)	$W=0=\frac{d}{dx}\left(f(x)\frac{d^{2}W}{dx^{2}}\right)+\eta^{2}{\left(\frac{r_{0}}{L}\right)}^{2}g(x)\frac{dW}{dx}$	Case (iii)

It can be seen from equations (3.1) and (3.2) that

3.5 $$\frac{dW}{dx}=0\;\text{and}\;W=0\;\Leftrightarrow \;\frac{dU}{dx}=0\;\text{and}\;U=0.$$

Thus, the clamped end condition for a beam is preserved by the above transformation for any functions *m*,*g* and *f*.

The free and pinned boundary configuration depends on how the shearing force (*V*) and bending moment (*M*) transform. For the harmonic vibration of Rayleigh beams, *M* and *V* are given by

3.6 $$M=f(x)\frac{d^{2}W}{dx^{2}}\;\mathrm{and}\;V=\frac{d}{dx}\left(f(x)\frac{d^{2}W}{dx^{2}}\right)+\eta^{2}{\left(\frac{r_{0}}{L}\right)}^{2}g(x)\frac{dW}{dx}.$$

Applying the transformation to equation (3.6), we have

3.7 $$f(x)\frac{d^{2}W}{dx^{2}}=\frac{d^{2}U}{dz^{2}}\frac{1}{\left(p)(q\right)}+U\left(p\frac{d^{2}q}{dz^{2}}+\frac{dp}{dz}\frac{dq}{dz}\right)\frac{1}{{\left(p\right)}^{2}{\left(q\right)}^{2}}+\frac{dU}{dz}\left(2p\frac{dq}{dz}+q\frac{dp}{dz}\right)\frac{1}{{\left(p\right)}^{2}{\left(q\right)}^{2}}$$

and

3.8 $$\begin{array}{rl} & \frac{d}{dx}\left(f(x)\frac{d^{2}W}{dx^{2}}\right)+\eta^{2}{\left(\frac{r_{0}}{L}\right)}^{2}g(x)\frac{dW}{dx}=\frac{d^{3}U}{dz^{3}}\frac{1}{q}+\frac{dq}{dz}\frac{d^{2}U}{dz^{2}}\frac{1}{{\left(q\right)}^{2}}+\frac{dU}{dz}(pq\left(3p\frac{d^{2}q}{dz^{2}}-2\frac{dp}{dz}\frac{dq}{dz}\right)\\ & \;+{\left(q\right)}^{2}\left(p\frac{d^{2}p}{dz^{2}}-2{\left(\frac{dp}{dz}\right)}^{2}\right)-4{\left(p\right)}^{2}{\left(\frac{dq}{dz}\right)}^{2})\frac{1}{{\left(p\right)}^{2}{\left(q\right)}^{3}}+U(q\left(p\left(p\frac{d^{3}q}{dz^{3}}+\frac{d^{2}p}{dz^{2}}\frac{dq}{dz}\right)-2{\left(\frac{dp}{dz}\right)}^{2}\frac{dq}{dz}\right)\\ & \;-2p\frac{dq}{dz}\left(p\frac{d^{2}q}{dz^{2}}+\frac{dp}{dz}\frac{dq}{dz}\right))\frac{1}{{\left(p\right)}^{2}{\left(q\right)}^{3}}+\eta^{2}\left({\left(\frac{r_{0}}{L}\right)}^{2}U\frac{dq}{dz}\frac{1}{{\left(q\right)}^{2}}+{\left(\frac{r_{0}}{L}\right)}^{2}\frac{dU}{dz}\frac{1}{q}\right). \end{array}$$

Free boundary configuration is preserved if the following relations hold:

3.9*a* $$f(x)\frac{d^{2}W}{dx^{2}}=0\;\Leftrightarrow \;\frac{d^{2}U}{dz^{2}}=0$$

and

3.9*b* $$\frac{d}{dx}\left(f(x)\frac{d^{2}W}{dx^{2}}\right)+\eta^{2}{\left(\frac{r_{0}}{L}\right)}^{2}g(x)\frac{dW}{dx}=0\;\Leftrightarrow \;\frac{d^{3}U}{dz^{3}}+\eta^{2}{\left(\frac{r_{0}}{L}\right)}^{2}\frac{dU}{dz}=0.$$

For equation (3.9a) to hold, the coefficients of *U* and d*U*/d*z* in equation (3.7) should be zero. That the coefficient of *U* equals zero implies (*p*(d^2^*q*/d*z*^2^)+(d*p*/d*z*)(d*q*/d*z*))=(*d*/d*z*)(*p*(d*q*/d*z*)) is zero. This holds when *p*(d*q*/d*z*) is a constant. For the coefficient of d*U*/d*z* to be zero, (2*pq*(d*q*/d*z*)+*q*^2^(d*p*/d*z*))=(*d*/d*z*)(*pq*^2^) should be zero. This holds when *pq*^2^ is a constant. Thus, when *p*(d*q*/d*z*) and *pq*^2^ are constants, equation (3.9a) holds. Equation (3.9b) is satisfied only when *p* and *q* are constants. This transforms a given uniform beam to a different uniform beam. When *p*(d*q*/d*z*) and *pq*^2^ are constants, equation (3.9b) will have an extra term in the right-hand side of the relation, which will add an error in the calculation of natural frequencies.

## 4. Finite-element validation

We use the FEM to compute the natural frequencies of the non-uniform beams. A thorough description of the methodology is available in standard textbooks [19]. In the finite-element formulation, the beam is discretized into many finite elements of equal length (*l*) each of which has two nodes, and each node has two degrees of freedom—transverse displacement (*w*) and the slope of deflection. Specifically, *w*, along the *i*th element, is given by

4.1 $$w=H_{1}W_{i}+H_{2}\phi_{i}+H_{3}W_{i+1}+H_{4}\phi_{i+1}=\lfloor H\rfloor {\left\{d\right\}}_{i}.$$

The Hermite shape functions (*H*_1_, *H*_2_, *H*_3_ and *H*_4_) are given by *H*_1_=2*ζ*^3^−3*ζ*^2^+1,*H*_2_=(*ζ*^3^−2*ζ*^2^+*ζ*)/*l*,*H*_3_=−2*ζ*^3^+3*ζ*^2^ and *H*_4_=(*ζ*^3^−*ζ*^2^)/*l*, where *ζ*=(*x*−*x*_*i*_)/*l*. The expressions for the kinetic energy *T*_*i*_ and potential energy *U*_*i*_ of the *i*th beam element are given by

4.2*a* $$T_{i}=\frac{1}{2}\int_{x_{i}}^{x_{i+1}}m(x){\left(\frac{\partial w}{\partial t}\right)}^{2}\;dx+\frac{1}{2}\int_{x_{i}}^{x_{i+1}}g(x){\left(\frac{\partial^{2}w}{\partial t\partial x}\right)}^{2}dx$$

and

4.2*b* $$U_{i}=\frac{1}{2}\int_{x_{i}}^{x_{i+1}}f(x){\left(\frac{\partial^{2}w}{\partial x^{2}}\right)}^{2}\;dx.$$

The elemental mass and stiffness matrices are given by

4.3*a* $$M_{ij}=\int_{x_{i}}^{x_{i+1}}g(x)\;H_{i}^{\prime }H_{j}^{\prime }\;dx+\int_{x_{i}}^{x_{i+1}}m(x)\;H_{i}H_{j}\;dx$$

and

4.3*b* $$K_{ij}=\int_{x_{i}}^{x_{i+1}}f(x)H_{i}^{\prime \prime }H_{j}^{\prime \prime }\;dx.$$

These elemental mass *M*_*ij*_ and stiffness *K*_*ij*_ matrices are assembled appropriately to obtain global mass [*M*] and stiffness [*K*] matrices. The natural frequencies (*η*) and mode shapes (*x*) are then obtained by solving the following eigenvalue problem:

4.4 $$[K]\{x\}=\eta^{2}[M]\{x\}.$$

The non-dimensional frequencies (*η*) of the uniform and the non-uniform Rayleigh beams are calculated for the following boundary configurations: (i) clamped–pinned, (ii) pinned–pinned and (iii) clamped–clamped. For the non-uniform beam, *f*,*g* and *m* obtained from the case *pq*^2^=*c* are chosen for clamped–pinned and pinned–pinned configurations. The frequencies obtained are listed in tables 2 and 3. For clamped–clamped configuration, the *f*,*g* and *m* from any of the four cases can be considered. The frequencies for this configuration are listed in table 4.

**Table 2.** Non-dimensional frequencies of a clamped–pinned beam (500 elements).

uniform beam $\left[\frac{r_{0}}{L}=0.09\right]$	isospectral non-uniform beam $\left[\frac{r_{0}}{L}=0.09\right]$
14.745	14.745
43.0405	43.0405
78.549	78.549
116.69	116.69
155.383	155.383
193.87	193.87

**Table 3.** Non-dimensional frequencies of a pinned–pinned beam (500 elements).

uniform beam $\left[\frac{r_{0}}{L}=0.09\right]$	isospectral non-uniform beam $\left[\frac{r_{0}}{L}=0.09\right]$
9.49728	9.49728
34.3645	34.3645
67.7395	67.7395
104.602	104.602
142.489	142.489
180.426	180.426

**Table 4.** Non-dimensional frequencies of a clamped–clamped beam (500 elements).

uniform beam $\left[\frac{r_{0}}{L}=0.09\right]$	isospectral non-uniform beam $\left[\frac{r_{0}}{L}=0.09\right]$
21.322	21.322
52.5959	52.5959
90.0704	90.0704
129.325	129.325
168.719	168.719
207.675	207.675

**Figure 10.**
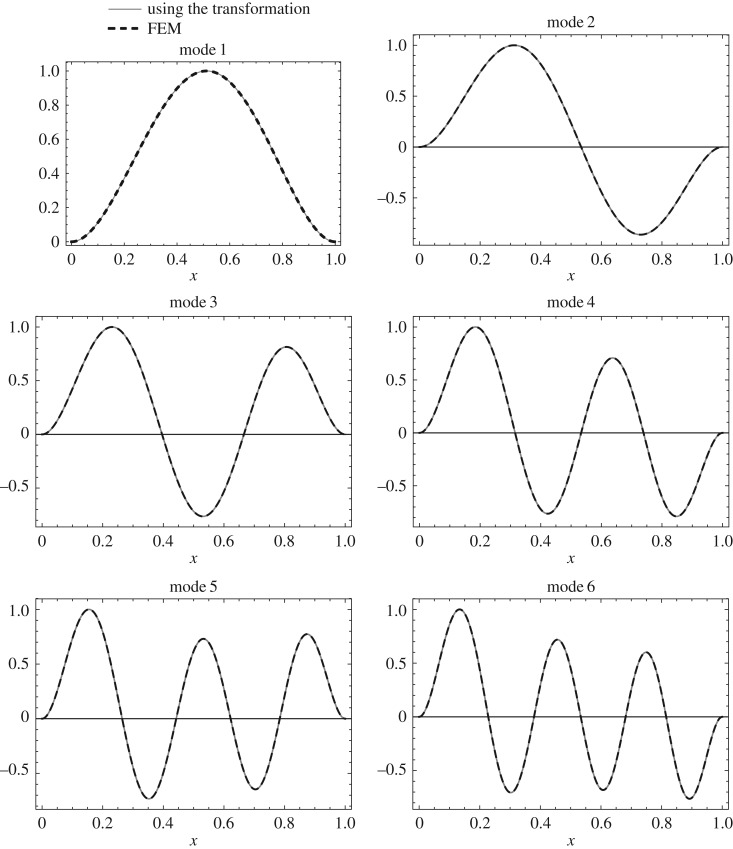
First six mode shapes calculated using the above transformation and FEM (500 elements) for the clamped–clamped non-uniform beam (Case 2: *α*=0.3, *β*=0.1).

**Figure 11.**
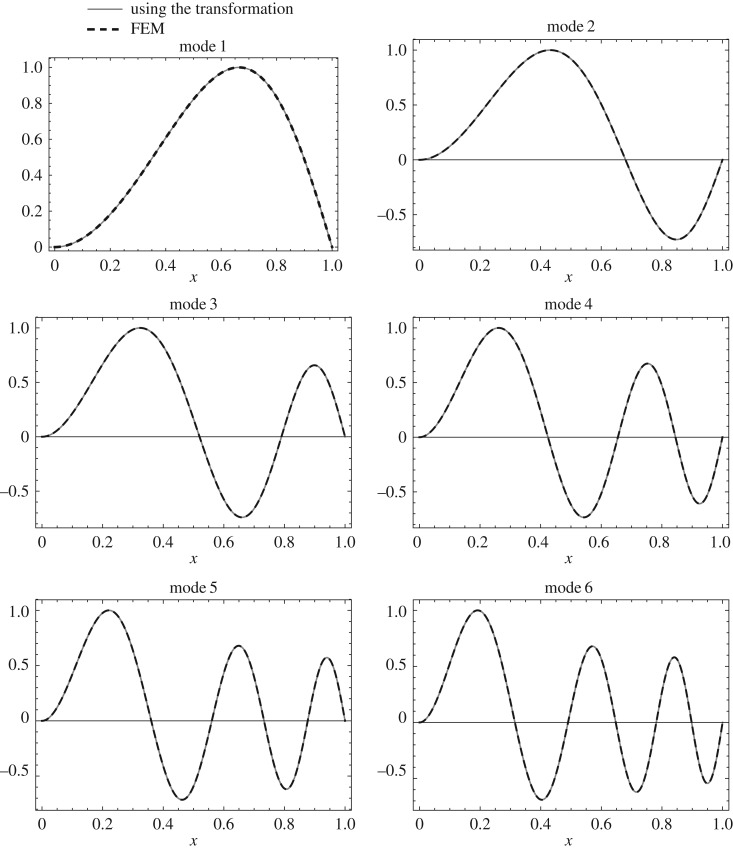
First six mode shapes calculated using the above transformation and FEM (500 elements) for the clamped–pinned non-uniform beam (Case 3: *c*=0.25, *α*=−1.56155).

**Figure 12.**
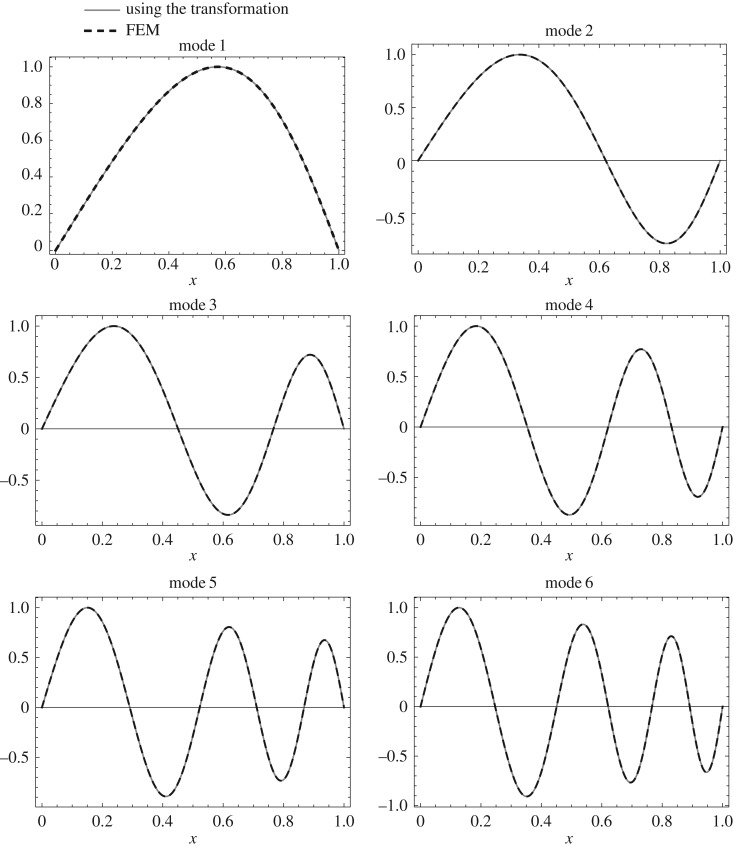
First six mode shapes calculated using the above transformation and FEM (500 elements) for the pinned–pinned non-uniform beam (Case 3: *c*=0.25, *α*=−1.56155).

The mode shape *U*(*z*) of the uniform clamped–clamped Rayleigh beam is given in appendix A. The mode shape *W*(*x*) of the isospectral non-uniform Rayleigh beam can be calculated using the above transformation from *U*(*z*) as follows. First, calculate *W*(*z*) using *W*(*z*)=*q*(*z*)*U*(*z*). Then calculate *W*(*x*) from *W*(*z*) by substituting *z* in terms of *x*. The first six mode shapes calculated using the above transformation and the mode shapes calculated using FEM for the non-uniform clamped–clamped beam are shown in figure 10. Similarly, the first six mode shapes calculated for the non-uniform clamped–pinned and pinned–pinned beam are shown in figures 11 and 12.

## 5. Conclusion

In this study, an analytical procedure for determining non-uniform Rayleigh beams, which are isospectral to a uniform beam, is presented. A transformation is used to convert the non-uniform beam equation to a uniform beam equation. Analytical expressions for the mass, bending stiffness and mass moment of inertia of such non-uniform beams are derived considering four specific cases. We provide the necessary conditions, on the auxiliary variables, required to preserve the boundary configurations. Beams having a rectangular cross section are presented for all the four cases to show the application of this analysis. The breadth, height and the ratio of modulus and density variations of the rectangular cross section, with a span, of isospectral non-uniform beams, are obtained in our study. The non-dimensional frequencies of the obtained isospectral non-uniform beams are calculated using FEM, and they are found to be the same as that of the given uniform beam for a particular boundary condition.

It is easier to analyse uniform beams. Once the dynamic characteristics of a uniform beam are obtained, from the above technique, we can create a non-uniform beam of same spectra as that of a uniform beam and know its dynamic characteristics. Also, recent advancements in machining techniques—such as additive manufacturing and rapid prototyping [20,21]—facilitate the manufacturing of beams with known breadth, height and material property variation. Structural identification issues, such as damage and blockage identification, have been solved with the help of quasi-isospectral operators for rods and symmetric ducts [22,23]. We can develop similar transformation for various versions of beams which along with the quasi-isospectral operators are an integral part of damage and blockage identification. Finally, the procedure can also be extended to find isospectral uniform rotating beams, axially loaded uniform beams and tapered rotating beams.

## Data accessibility

Supporting data in this work can be accessed via the following link: https://doi.org/10.5061/dryad.154dj [24]. Not applicable for the tabular data: the paper contains sufficient detail to reproduce these results.

## Appendix A. Exact solution and the mode shape

The exact solution of the uniform Rayleigh beam equation is as follows:

A 1 $$\begin{array}{rl} U(z) & =c_{1}e^{z\sqrt{-\eta \sqrt{(r_{0}/L{)}^{4}\eta^{2}+4}-(r_{0}/L{)}^{2}\eta^{2}}/\sqrt{2}}+c_{2}e^{-z\sqrt{-\eta \sqrt{(r_{0}/L{)}^{4}\eta^{2}+4}-(r_{0}/L{)}^{2}\eta^{2}}/\sqrt{2}}\\ & \;+c_{3}e^{z\sqrt{\eta \sqrt{(r_{0}/L{)}^{4}\eta^{2}+4}-(r_{0}/L{)}^{2}\eta^{2}}/\sqrt{2}}+c_{4}e^{-z\sqrt{\eta \sqrt{(r_{0}/L{)}^{4}\eta^{2}+4}-(r_{0}/L{)}^{2}\eta^{2}}/\sqrt{2}}. \end{array}$$

Applying the boundary conditions for the clamped–clamped non-uniform beam, we arrive at a system of equations. Upon equating the determinant of the coefficient matrix to zero, we obtain

A 2 $$\begin{array}{rl} & {\left(\frac{r_{0}}{L}\right)}^{2}\eta \sin\left(\frac{\sqrt{\eta (\sqrt{{\left(r_{0}/L\right)}^{4}\eta^{2}+4}+{\left(r_{0}/L\right)}^{2}\eta )}}{\sqrt{2}}\right)\sinh\left(\frac{\sqrt{2}}{\sqrt{\sqrt{{\left(r_{0}/L\right)}^{4}\eta^{2}+4}\eta +{\left(r_{0}/L\right)}^{2}}}\right)\\ & \;+2\cos\left(\frac{\sqrt{\eta (\sqrt{{\left(r_{0}/L\right)}^{4}\eta^{2}+4}+{\left(r_{0}/L\right)}^{2}\eta )}}{\sqrt{2}}\right)\cosh\left(\frac{\sqrt{2}}{\sqrt{\sqrt{{\left(r_{0}/L\right)}^{4}\eta^{2}+4}\eta +{\left(r_{0}/L\right)}^{2}}}\right)-2=0. \end{array}$$

Solving the above equation, we obtain the natural frequencies of the uniform clamped–clamped Rayleigh beam. To arrive at the *i*th mode shape we substitute the *i*th natural frequency in equation (5.1) and solve for *c*_1_, *c*_2_, *c*_3_ and *c*_4_. The values of *c*_1_, *c*_2_, *c*_3_ and *c*_4_ for *η*=90.0704 (third mode) are as follows:

A 3 $$c_{1}=-0.500178-0.349711i,c_{2}=-0.500178+0.349711i,c_{3}=0.0003567,c_{4}=1.$$
