# Correction to ‘Isospectrals of non-uniform Rayleigh beams with respect to their uniform counterparts’

**DOI:** 10.1098/rsos.180414

**Published:** 2018-04-11

**Authors:** Srivatsa Bhat K, Ranjan Ganguli

*R. Soc. open sci.* 171717. (Published Online 14 February 2018). (doi:10.1098/rsos.171717)

This correction concerns equation 2.17 and consequently figs. 1 and 2 of [1]. The correct equation, and figures 1 and 2 are as follows:

1 $$\{f(x),g(x),m(x)\}=\left\{\frac{{\left(\beta^{2}+2\alpha x\right)}^{3/2}}{c_{1}^{2}},\frac{\sqrt{\beta^{2}+2\alpha x}}{c_{1}^{2}},\frac{1}{c_{1}^{2}\sqrt{\beta^{2}+2\alpha x}}\right\}$$

**Figure 1.**
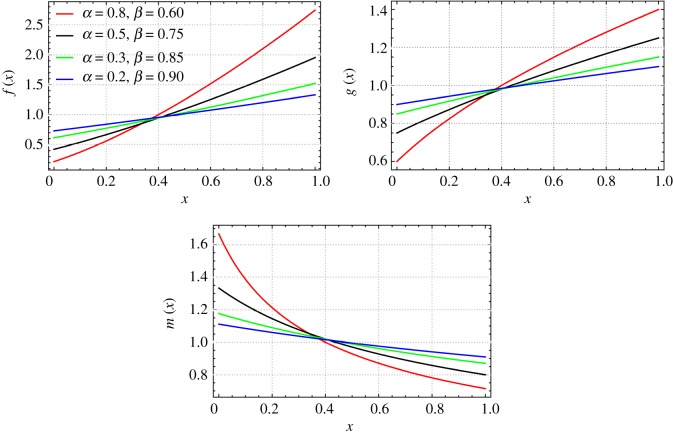
Mass, bending stiffness and mass moment of inertia functions of a non-uniform beam isospectral to a uniform beam (Case-1: *β* = {0.60, 0.75, 0.85, 0.90}, *c*_1_ = 1 and *r*_0_/*L* = 0.09).

**Figure 2.**
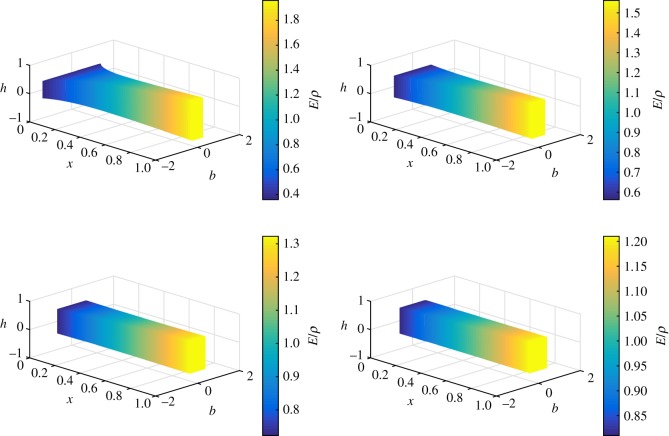
Breadth, height and ratio of modulus and density distributions of non-uniform beams isospectral to a uniform beam (Case-1: *β*={0.60,0.75,0.85,0.90}, *c*_1_=1 and *r*_0_/*L*=0.09).
